# Cervicovaginal Microbiota Predicts *Neisseria gonorrhoeae* Clinical Presentation

**DOI:** 10.3389/fmicb.2021.790531

**Published:** 2022-02-10

**Authors:** Angela Lovett, Arlene C. Seña, Andrew N. Macintyre, Gregory D. Sempowski, Joseph A. Duncan, Andreea Waltmann

**Affiliations:** ^1^Department of Pharmacology, University of North Carolina at Chapel Hill, Chapel Hill, NC, United States; ^2^Division of Infectious Diseases, Department of Medicine, University of North Carolina at Chapel Hill, Chapel Hill, NC, United States; ^3^Institute for Global Health and Infectious Diseases, University of North Carolina at Chapel Hill, Chapel Hill, NC, United States; ^4^School of Medicine and Duke Human Vaccine Institute, Duke University, Durham, NC, United States; ^5^Lineberger Comprehensive Cancer Center, University of North Carolina at Chapel Hill, Chapel Hill, NC, United States

**Keywords:** *Neisseria gonorrhoeae*, bacterial vaginosis, *Lactobacillus*, microbiome, symptoms

## Abstract

*Neisseria gonorrhoeae* infection of the female lower genital tract can present with a spectrum of phenotypes ranging from asymptomatic carriage to symptomatic cervical inflammation, or cervicitis. The factors that contribute to the development of asymptomatic or symptomatic infections are largely uncharacterized. We conducted a pilot study to assess differences in the cervicovaginal microbial community of patients presenting with symptomatic vs. asymptomatic *N. gonorrhoeae* infections to a sexually transmitted infections (STI) clinic. DNA was isolated from cervicovaginal swab specimens from women who tested positive for *N. gonorrhoeae* infection using a clinical diagnostic nucleic acid amplification test. We performed deep sequencing of 16S ribosomal RNA gene amplicons, followed by microbiome analyses with QIIME, and species-specific real-time PCR to assess the composition of microbial communities cohabitating the lower genital tract with the infecting *N. gonorrhoeae*. Specimens collected from asymptomatic individuals with *N. gonorrhoeae* infection and no co-infection with *Chlamydia trachomatis* and/or *Trichomonas vaginalis* carried *Lactobacillus-*dominant microbial communities more frequently than symptomatic patients without co-infection. When compared to asymptomatic individuals, symptomatic women had microbial communities characterized by more diverse and heterogenous bacterial taxa, typically associated with bacterial vaginosis (BV) [*Prevotella*, *Sneathia*, *Mycoplasma hominis*, and Bacterial Vaginosis-Associated Bacterium-1 (BVAB1)/“*Candidatus Lachnocurva vaginae*”]. Both symptomatic and asymptomatic *N. gonorrhoeae* patients with additional STI co-infection displayed a BV-like microbial community. These findings suggest that *Lactobacillus-*dominant vaginal microbial community may protect individuals from developing symptoms during lower genital tract infection with *N. gonorrhoeae*.

## Introduction

*Neisseria gonorrhoeae* is a sexually transmitted bacterial pathogen responsible for 90 million infections globally each year (Rowley et al., 2019). *N. gonorrhoeae* is a strictly human pathogen and infections are typically localized to the lower genital tract. Although acute symptomatic infection is often recognized and treated with antibiotic therapy, a surprisingly large proportion of *N. gonorrhoeae* infections are asymptomatic (Handsfield et al., 1974; Platt et al., 1983; Sandstrom et al., 1984). Upwards of 50% of lower genital tract infections in females are asymptomatic (Kent et al., 2005; Lovett and Duncan, 2018). While the prevailing dogma is that male urethral infections are symptomatic, compelling reports have documented that asymptomatic genital gonorrhea is prevalent in both biological sexes and across a wide range of settings (Handsfield et al., 1974; Potterat et al., 1987; Sherrard and Barlow, 1996; Hazel et al., 2014; Ong et al., 2017; Martin-Sanchez et al., 2020). Asymptomatic infection is not without consequence. Untreated asymptomatic infections can ascend to the upper genital tract leading to health complications including pelvic inflammatory disease and infertility in women (Wiesenfeld et al., 2012; Reekie et al., 2018, 2019). Asymptomatic genital *N. gonorrhoeae* poses a risk of onward transmission to sex partners and ascending infection. Mathematical models of gonorrhea transmission have confirmed unequivocally the significant contribution of subclinical infections in maintaining community transmission (Hazel et al., 2015). The determinants and mechanisms that underlie asymptomatic and symptomatic gonorrhea infection are unknown. This knowledge gap greatly hinders efforts to develop new strategies for gonorrhea control.

Symptomatic *N. gonorrhoeae* infection most commonly leads to localized host inflammation at the site of infection, urethritis in males, and cervicitis in females. *N. gonorrhoeae* itself is resistant to many host antimicrobial responses, which may contribute to its ability to cause infection under these conditions of localized inflammation. During an infection, *N. gonorrhoeae* must compete with the natural microbial community at the mucosal surface to establish infection (Aroutcheva et al., 2001). Cervicovaginal microbial communities play an important role in sexual and reproductive outcomes, including protection from pathogens, as the composition of the cervicovaginal microbiota has been shown to modify susceptibility to several sexually transmitted pathogens (STI) (Sha et al., 2005; Coleman et al., 2007; Atashili et al., 2008; Masson et al., 2014, 2015; Anahtar et al., 2015; McClelland et al., 2018). For example, human vaginal microbiota dominated by *Lactobacillus crispatus* is associated with reduced risk of acquisition of STI, like HIV (Borgdorff et al., 2014; Gosmann et al., 2017; van der Veer et al., 2017; Tamarelle et al., 2019). In addition, women with clinically apparent bacterial vaginosis (BV), a clinical condition characterized by depleted levels of *Lactobacillus* species and an increased abundance of diverse groups of facultative anaerobes, have an increased risk of acquiring and transmitting STI, including *N. gonorrhoeae* (Wiesenfeld et al., 2003; Gallo et al., 2012; Borgdorff et al., 2014; Bautista et al., 2017) and at an increased risk of adverse reproductive and obstetric outcomes (Brotman, 2011; Elovitz et al., 2019; Fettweis et al., 2019), irrespective of whether the BV is symptomatic or not (Atashili et al., 2008; Brotman, 2011; McKinnon et al., 2019). These factors indicate a mechanistic contribution of *L. crispatus* to protection from STI, presumably through the production of lactic acid and thus the maintenance of a low-pH vaginal microenvironment (Tuddenham et al., 2021). The reasons why the microbiota of some women is dominated by *Lactobacillus* species, whereas that of others becomes dominated by anaerobes are not completely understood.

Molecular definitions of the cervicovaginal microbiota of reproductive age women, with respect to microbial composition and diversity, are now standard (Ravel et al., 2011; Anahtar et al., 2015; Gosmann et al., 2017). Using 16S rRNA sequencing or metagenomics, distinct cervicovaginal microbial community types (CTs) have been classified (Ravel et al., 2011; Anahtar et al., 2015; Gosmann et al., 2017). The “optimal types” CT1 and CT2 are dominated by *L. crispatus* and *L. gasseri*, respectively. The other two *Lactobacillus*-dominant molecular community types are *L. iners* (CT3) and *L. jensenii* (CT5). Notably, not all *Lactobacillus*-dominant types are equal in their protective effect against STI, as *L. iners*-dominated vaginal microbiotas may actually place patients at higher risk of STI infection, like chlamydia or HIV, when compared to *L. crispatus*-dominant microbiotas (Gosmann et al., 2017; van der Veer et al., 2017; Tamarelle et al., 2019). Finally, CT4 communities are characterized by a diverse and heterogenous group of anaerobes (e.g., *Atopobium, Prevotella, Dialister, Gardnerella, Megasphaera, Peptoniphilus, Sneathia, Eggerthella, Aerococcus, Finegoldia*, and *Mobiluncus;* Ravel et al., 2011) and this vaginal environment is a significant risk factor for having clinically diagnosed BV (Amsel et al., 1983; Srinivasan et al., 2012; Peebles et al., 2019) and is termed molecular BV (McKinnon et al., 2019).

Studies examining the impact of vaginal microbiomes on inflammatory states found that women with clinical BV or molecular BV (i.e., CT4 microbial communities) had higher cervicovaginal levels of pro-inflammatory cytokines (IL-1α, IL-1β, IL-6, IL-12, and IL-8) than BV-negative or *Lactobacillus*-dominant women, respectively (Masson et al., 2014; Gosmann et al., 2017; Joag et al., 2019). Notably, the more the vaginal microbiota shifts away from a state dominated by *L. crispatus* toward dysbiosis (i.e., toward CT3 and CT4 types), the more marked the inflammation (Cohen et al., 2010; Anahtar et al., 2015; Lennard et al., 2018), independently of concurrent STIs, including gonorrhea (Anahtar et al., 2015). This indicates that the variations in vaginal microbial diversity that are common in women with BV could influence inflammatory responses that characterize symptomatic *N. gonorrhoeae* infection.

We sought to understand whether differences in microbial composition of the genital tract were associated with symptomatic or asymptomatic presentation of *N. gonorrhoeae* infection, by utilizing 16S ribosomal RNA (rRNA) amplicon deep sequencing of clinician-collected cervicovaginal specimens from females diagnosed with gonorrhea.

## Materials and Methods

### Study Population and Sample Collection

Specimens used in this study were remnant specimens collected from female patients that attended a public STI clinic located in Durham, North Carolina in 2011 and tested positive for *N. gonorrhoeae* using the clinical diagnostic assay Aptima Combo 2^®^ assay (for CT/NG) by Hologic. Clinician-collected cervicovaginal swabs used for diagnosis were collected as per routine care prior to any treatment. The age, race, reported symptoms, and diagnosis for each study subject were linked to each specimen by a study clinician when a positive specimen was identified. The de-identified remnant cervical swab samples were stored in Aptima buffer in their respective transport tubes at −80°C until DNA extraction was performed.

### DNA Extraction

Two hundred microliters of Aptima buffer from each sample were transferred to sterile 2-ml tubes containing 200 mg of ≤100-μm glass beads (Sigma), 0.3 ml of 20 mg/ml lysozyme solution (Thermo Fisher), and 0.3 ml of Qiagen ATL buffer. Bead-beating was then carried out for 10 min in a Qiagen TissueLyser II at 30 Hz to ensure optimal DNA yield from Gram-positive bacteria. Subsequently, samples were incubated at 37°C for 30 min. After a brief centrifugation, supernatants were aspirated and transferred to a new sterile tube with Qiagen AL buffer containing Proteinase K (600 IU/μl). Samples were then incubated at 70°C for 10 min. DNA was purified using a standard on-column purification method using Zymo-spin mini columns and Qiagen buffers AW1 and AW2 as washing agents. DNA was eluted in 100 μl of 10 mM Tris (pH 8.0).

### 16S Ribosomal RNA Gene Sequencing

For amplicon library preparation, we used fusion primers composed of Ion Torrent adapter 5′-*CCATCTCATCCCTGCGTGTCTCCGACTCAG*-3′ for the forward primer and 5′-*CCTCTCTATGGGCAGTCGGTGAT*-3′ for the reverse primer, and universal bacterial primer 8F 5′-*AGAGTTTGATCCTGGCTCAG*-3′ and 338R 5′-*GCTGCCTCCCGTAGGAGT*-3′. The forward primer also included a 10-bp IonXpress™ barcode, unique to each sample. Each bacterial DNA sample was run in duplicate in a 25-μl PCR reaction containing: 4 μl of 5 × MyTaq Reaction Buffer (Bioline); 0.6 μl each of 15 μM Forward Primer and 15 μM Reverse Primer (Integrated DNA Technologies); 0.5 μl of MyTaq HS DNA Polymerase (Bioline); 100 ng of template DNA; and water to 25 μl. Samples were denatured at 94°C for 5 min, followed by 35 cycles of 94°C for 45 s, 55°C for 45 s, and 72°C for 90 s, followed by an extension at 72°C for 10 min and a 4°C hold. Each sample was visualized on a 2% agarose gel. Bands were excised and duplicate bands were combined into one tube. Gel purification was performed using the Qiagen Gel Extraction Kit (Qiagen) according to the manufacturer’s protocol. Samples were quantified using an Agilent 2100 Bioanalyzer (Agilent). Quantification information was used to create a library by combining equimolar concentrations of each sample. The prepared library was sequenced on the Ion Torrent PGM Instrument (Life Technologies) according to the manufacturer’s protocol at UNC-CH High Throughput Sequencing Facility.

### Sequence Data Analysis

Sequencing output was demultiplexed and the resulting paired-end reads were joined using the QIIME 1.9.0 (Caporaso et al., 2010b) by invocation of fastq-join with the default parameters. Index and linker primer sequences were trimmed, and the reads were subsequently filtered for quality using a sliding window of 50 bases, moving by five bases, requiring an average quality score of 20 or above. Quality control of both raw and processed sequencing reads was verified by FastQC (FastQC, 2015). Sequences were clustered into operational taxonomic units (OTU) based on the *de novo* OTU picking algorithm using the QIIME implementation of UCLUST (Edgar, 2010) at a similarity threshold of 97%. OTUs identified as chimeric by vsearch (Rognes et al., 2016) of the ChimeraSlayer “gold” reference database (Haas et al., 2011) and those composed of a single read (singletons) were eliminated. The remaining OTUs were assigned taxonomic identifiers with respect to the Greengenes database (DeSantis et al., 2006), and their sequences were aligned using template alignment through PyNAST (Caporaso et al., 2010a), and a phylogenetic tree was built with FastTree 2.1.3 (Price et al., 2010). If after the Greengenes taxonomic assignment a taxon of interest was ambiguous at the genus level or when putative species taxonomy was sought, we consulted the 16S sequences in the National Center for Biotechnology Information (NCBI) GenBank repository with the Basic Local Alignment Search Tool (BLAST) (Altschul et al., 1990) and/or with the multiple sequence comparison by log-expectation method (MUSCLE) implemented in Geneious using reference genomes.

### Microbiome Analyses

Alpha diversity was measured by three different metrics (Chao1; and observed species; phylogenetic diversity, PD) using QIIME. Beta diversity estimates were calculated within QIIME using weighted and unweighted Unifrac distances (Lozupone and Knight, 2005) between samples. Results were summarized and visualized through principal coordinate analysis (PCoA) in QIIME. Cervicovaginal community types (CTs) were assigned using established definitions (Ravel et al., 2011; Anahtar et al., 2015; Gosmann et al., 2017) based on diversity and relative abundance of bacterial taxa. These definitions classify samples with relative majority abundance assigned to *L. crispatus*, *L. gasseri*, *L. iners*, or *L. jensenii* as CT1, CT2, CT3, and CT5, respectively. According to the same definitions, low *Lactobacillus* communities comprising a diverse and heterogenous group of anaerobes (e.g., *Atopobium, Prevotella, Dialister, Gardnerella, Megasphaera, Peptoniphilus, Sneathia, Eggerthella, Aerococcus, Finegoldia*, and *Mobiluncus* are classified as CT4 or “molecular bacterial vaginosis”) (McKinnon et al., 2019).

### Microbial DNA qPCR Array

The Vaginal Flora Microbial DNA qPCR Array (Cat. no. 330261 BAID-1902Y, Qiagen) was used for vaginal microbiome profiling. Each specimen was processed and run on the 96-well array plate format. The array contained assays for the detection of 90 microbial species, 2 Pan bacteria controls (Pan Bacteria 1, Pan Bacteria 3), 1 Pan Fungi control (Pan *Aspergillus/Candida*), 2 host controls (Hs/Mm.GAPDH Hs/Mm.HBB1), and a positive PCR control (PPC). Five hundred nanograms of genomic DNA was mixed with 1,275 μl of microbial qPCR Mastermix and water as needed to bring the total volume to 2,550 μl, using the manufacturer’s instructions. Individual reaction mix aliquots of 25 μl were added to each well of the plate, and the array plate was tightly sealed, centrifuged at 1,000 rpm for 1 min, and loaded onto the Real-Time PCR machine. PCR was performed with an initial PCR activation step at 95°C for 10 min, followed by 2-step cycling of denaturation for 15 s at 95°C, with annealing and extension for 2 min at 60°C for 40 cycles. The CT values for each well were imported into the Microbial DNA qPCR Array Excel template (Qiagen) for analysis. The ΔCT between the patient sample DNA and no DNA template negative control was calculated for each set of species-specific primer sets, following manufacturer’s instructions for analysis. A species was reported as “present, +” if ΔCT was >3, an eightfold increase in signal over background.

### Statistical Analyses

Statistical analyses were performed in PRISM 9 or STATA 16. Differences in donor characteristics and between sample groups were investigated using the Fisher’s exact test. We compared α-diversity between sample groups with non-parametric two-sample *t-*tests using 1,000 Monte Carlo permutations to calculate the *p*-values. To test whether sample groups were statistically different, we used non-parametric ANOSIM (ANalysis Of Similarities) tests and non-parametric two-sample *t*-tests with 1,000 Monte Carlo permutations to derive *p*-values implemented in QIIME. For comparisons between multiple groups, one-way ANOVA was used, correcting for multiple comparisons with Tukey *post hoc* tests.

## Results

### Cohort Characteristics of *Neisseria gonorrhoeae*-Infected Women

In this pilot study, we used remnant nucleic acid material from cervical swabs collected from a convenience sample of 19 women deemed to be *N. gonorrhoeae-*positive by Aptima clinical diagnostic testing. Of these, ten individuals reported symptoms to the provider (defined as symptomatic) and nine did not (defined as asymptomatic) (Table 1). The reported symptoms at the time of cervical swab sampling included vaginal discharge (9/10, 90.0%), genital irritation (1/10, 10.0%), and dysuria (2/10, 20%). There was a trend for younger women to report symptoms (*p* = 0.090). Of the 19 specimens, 17 (89.5%) were collected from women who identified as having African American race. Among the African American women, the proportion of symptomatic and asymptomatic individuals was comparable (*p* = 0.211). The presence or absence of *C. trachomatis*, another STI pathogen, was assessed by NAAT testing in conjunction with clinical *N. gonorrhoeae* testing, and no difference in *C. trachomatis* prevalence was observed between asymptomatic and symptomatic presentation (*p* = 0.590).

**TABLE 1:** Study population characteristics at baseline.

Baseline characteristics	Symptomatic	Asymptomatic	*p*-value
		
	*n* = 10	*n* = 9	
Age, mean (range)	21.1 (15–37)	26.6 (16–40)	0.09
	Black (10)	Black (7)	n/a
Race (*n*)	Latina (0)	Latina (1)	
	White (0)	White (1)	
Symptoms (%)	Vaginal discharge (90.0)	None (100.0)	n/a
	Genital irritation (10.0)		
	Dysuria (20.0)		

*The age, race, and self-reported symptoms of 19 women positive for N. gonorrhoeae infection by clinical test who presented at a local STI clinic are provided.*

Since other STI pathogens could also be responsible for causing lower genital tract symptoms (e.g., *Trichomonas vaginalis* or *Mycoplasma genitalium*), we used a commercial microbial qPCR array to test for the presence of other STI pathogens (Table 2). Specimens from two women did not provide evaluable results due to insufficiently recovered DNA material. Among the 17 specimens that gave analyzable results on this array, 6 of 7 (85.7%) asymptomatic specimens and 8 of 10 (80.0%) symptomatic specimens had detectable *Neisseria* species DNA (Table 2). Because these specimens all tested positive for *N. gonorrhoeae* using the Aptima Combo 2, these results indicate that the sensitivity of the microbial qPCR array may be lower for detecting *N. gonorrhoeae* in this specimen type than the Aptima Combo 2. The technical sensitivity of the Aptima assay for detection of *N. gonorrhoeae* is reported as 50 colony-forming units (Aptima^®^ G-P), while the qPCR array is reported to be less than 100 copies of the 16S genomic target (Qiagen, 2021), which would correlate with 25 bacterial cells or colony-forming units. However, the potential interference of materials from clinical specimens is not documented for the qPCR array, which is not approved for clinical diagnostic purposes, while the published sensitivity and specificity of the Aptima assay for the detection of *N. gonorrhoeae* in clinical specimens are 97.7 and 99.0%, respectively (Hologic, 2016). *C. trachomatis* was detected in three of three specimens that were positive by Aptima Combo 2 test and had evaluable qPCR results in the qPCR array (Table 2). *T. vaginalis* was detected in 4 of 10 (40.0%) evaluable symptomatic individuals and 2 of 7 (28.6%) asymptomatic individuals (Table 2). *M. genitalium* was not detected by qPCR array in any of the specimens (Table 2). When accounting for both clinical *C. trachomatis* testing by Aptima Combo 2 and real-time PCR array results, the proportion of STI co-infection with *C. trachomatis* or *T. vaginalis* was not significantly different between symptomatic (5/10, 50.0%) and asymptomatic (5/9, 55.6%) individuals (*p* = 0.625). Because co-infection with other STI pathogens was not associated with symptomatic presentation, we next sought to assess whether the non-STI vaginal microbial community was associated with the presence or absence of symptoms.

**TABLE 2:** Results of clinical Aptima (NAAT) test results and microbial DNA real-time PCR (qPCR) array.

	*N. gonorrhoeae* only									*N. gonorrhoeae* and STI co-infections									
Clinical test	Asymptomatic				Symptomatic					Asymptomatic					Symptomatic				
Aptima GC	+	+	+	+	+	+	+	+	+	+	+	+	+	+	+	+	+	+	+
Aptima CT	–	–	–	–	–	–	–	–	–	–	+	+	+	–	–	+	–	+	–
**qPCR**																			
*N. gonorrhoeae*	+	+	+	+	+	+	–	+	+	+	+	n/a	n/a	–	+	+	–	+	+
*C. trachomatis*	–	–	–	–	–	–	–	–	–	–	+	n/a	n/a	–	–	+	–	+	–
*M. genitalium*	–	–	–	–	–	–	–	–	–	–	–	n/a	n/a	–	–	–	–	–	–
*T. vaginalis*	–	–	–	–	–	–	–	–	–	+	–	n/a	n/a	+	+	+	+	–	+

*Each column across the categories reflects one individual participant.*

*+, Detected; – not detected; n/a, insufficient sample for qPCR; GC, N. gonorrhoeae; CT, C. trachomatis.*

### *Neisseria* spp. Abundance Represents a Small Proportion of Bacterial Communities in Both Symptomatic and Asymptomatic Patients

**FIGURE 1:**
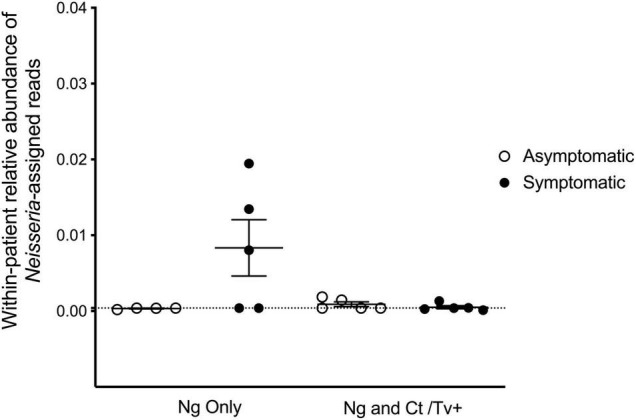
Reads assigned to the *Neisseria* genus comprise a small proportion of the cervicovaginal microbial community. We characterized the genital microbial communities of 19 females with clinically confirmed gonorrhea using 16S amplicon deep sequencing and microbiome analysis with QIIME. After demultiplexing and eliminating low-quality reads, 100,216 reads were retained for downstream analyses (mean number of reads per sample = 5,274; range = 2,470–9,528 reads). *Neisseria*-assigned 16S ribosomal RNA gene reads made up only 0.24% of all reads in the dataset and are plotted for each individual’s microbial community. No significant differences in relative abundance of 16S reads from *Neisseria* spp. between any group was observed using one-way ANOVA with Tukey’s correction for multiple comparisons.

The genital microbial community of the 19 study participants was characterized with 16S amplicon deep sequencing. A total 157,006 paired-end reads were obtained. After demultiplexing and elimination of low-quality reads, 100,216 reads were retained for downstream analyses of alpha diversity and beta diversity (mean number of reads per sample = 5,274; range = 2,470–9,528 reads). Paired reads were deposited in the Sequence Read Archive (SRA) under the accession PRJNA768436. The individual microbial communities of *N. gonorrhoeae*-infected patients were compared to those who presented with and without symptoms. Because other STI pathogens might be associated with different microbial community profiles, we also compared specimens from individuals without co-infecting *C. trachomatis* or *T. vaginalis* (by clinical test and/or real-time PCR) separately from those with co-infection. We first examined whether the relative abundance of *Neisseria* spp. assigned reads was associated with symptomatic presentation. *Neisseria*-assigned reads made up only 0.24% of all reads in the dataset and were a minor component of the bacterial community in each individual (Figure 1). In this limited set of specimens, the point estimate of the relative of abundance of reads from *Neisseria* spp. was highest in symptomatic individuals without *C. trachomatis* or *T. vaginalis* co-infection, though no significant difference in relative abundance between any group was observed (Figure 1).

### Microbial Community Diversity Is Different Between Symptomatic and Asymptomatic *Neisseria gonorrhoeae* Infection

**FIGURE 2:**
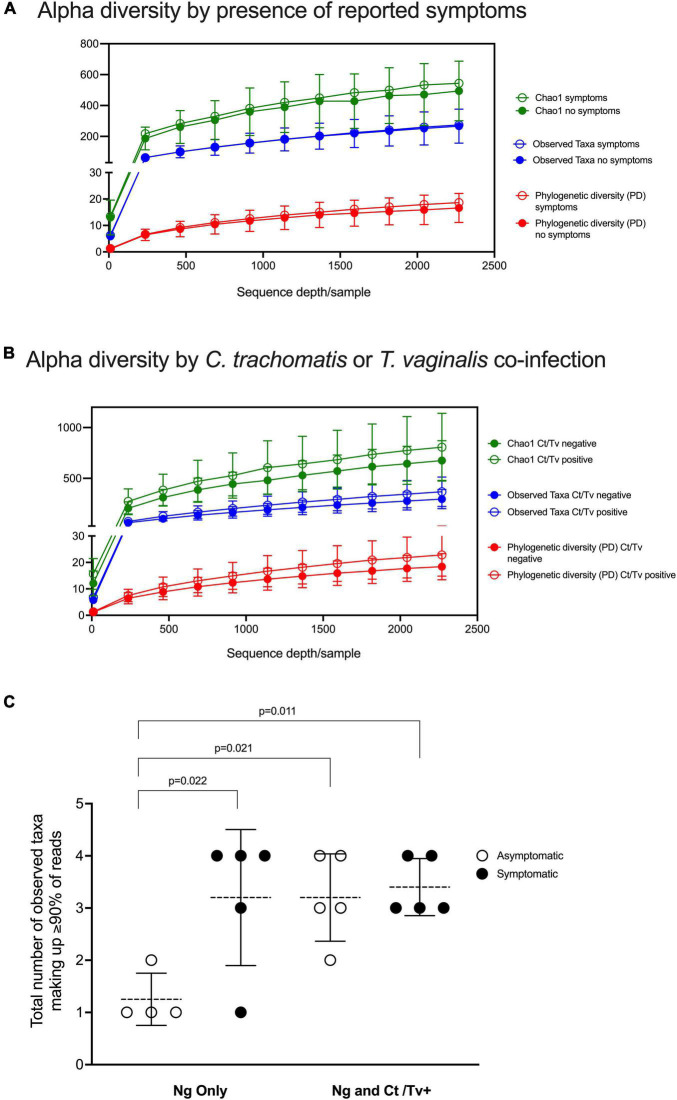
Alpha diversity analyses with respect to clinical presentation and other STI co-infection. Alpha diversity was measured by three different metrics (Chao1; observed species; and PD, phylogenetic diversity) at a depth of 2,270 reads and visualized as rarefaction plots. Overall alpha diversity are plotted for the symptomatic and asymptomatic *N. gonorrhoeae* infection patient groups **(A)** and for the co-infection patient groups (i.e. with and without other STI) **(B)**. The number of dominant taxa comprising the majority of the microbial community (i.e., 90% of all detected taxa) is plotted for individuals with asymptomatic and symptomatic *N. gonorrhoeae* infection with and without STI co-infection **(C)**. Statistically significant differences between patient groups were explored with one way ANOVA with Tukey’s correction for multiple comparisons.

The overall alpha diversity did not differ when observed taxa, Chao1, and phylogenetic diversity were compared between individuals with symptomatic and asymptomatic *N. gonorrhoeae* infection (Figure 2A and Supplementary Figures S1A–C) and between individuals with and without other STI (Figure 2B and Supplementary Figures S1D,E). However, the number of dominant taxa comprising the majority of the microbial community (i.e., 90% of all detected taxa) was significantly lower in individuals with asymptomatic *N. gonorrhoeae* infection without STI co-infection vs. both individuals with symptomatic *N. gonorrhoeae* and with STI co-infection (Figure 2C).

**FIGURE 3:**
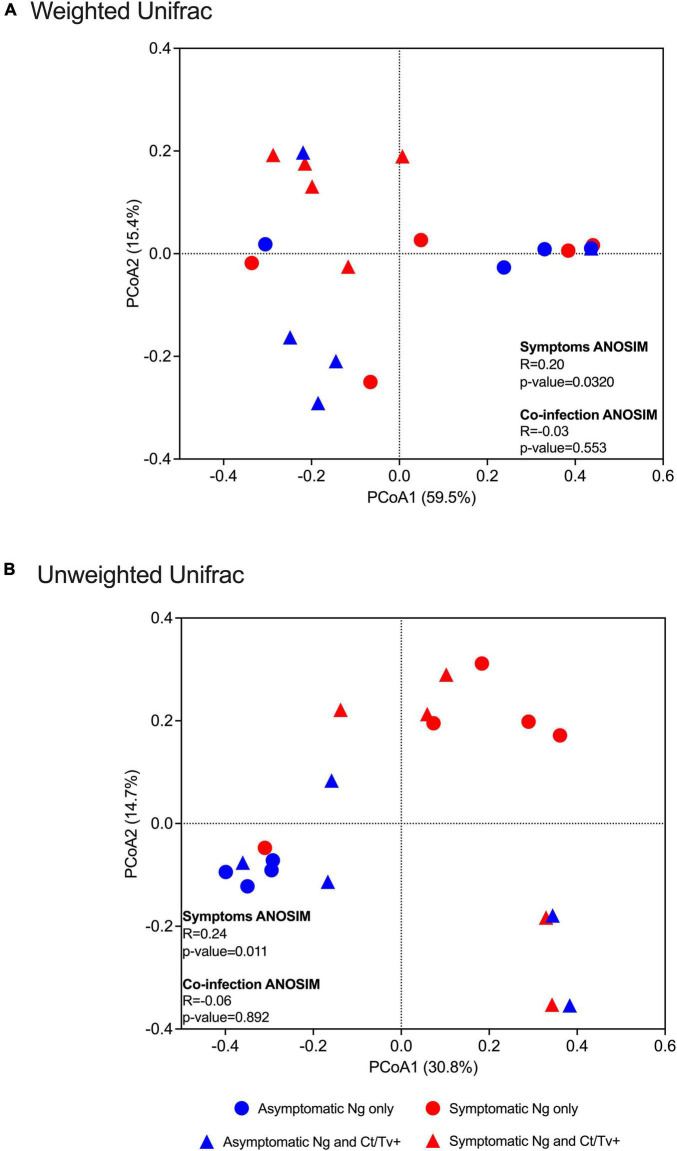
Beta diversity analyses with respect to clinical presentation and other STI co-infection. Beta diversity estimates were calculated within QIIME using weighted **(A)** and unweighted Unifrac **(B)** distances between samples. Results were summarized and visualized through principal coordinate analysis (PCoA) in QIIME. ANOSIM tests were used to assess the strength of the clustering patterns and statistical significance. Differences between symptomatic and asymptomatic patients, but not between patients with and without *C. trachomatis* and/or *T. vaginalis* co-infection, were found, with clear separation on PCoA plots by two different methods: weighted Unifrac (ANOSIM *R* = 0.20, *p*-value = 0.032, **A**) and unweighted Unifrac (ANOSIM *R* = 0.24, *p*-value = 0.011).

Differences between symptomatic and asymptomatic patients, but not between patients with and without *C. trachomatis* and/or *T. vaginalis* co-infection, were reflected in beta diversity analyses, with statistically significant ANOSIM tests and clear separation on PCoA plots by two different methods: weighted Unifrac (ANOSIM *R* = 0.20, *p*-value = 0.032, Figure 3A) and unweighted Unifrac (ANOSIM *R* = 0.24, *p*-value = 0.011, Figure 3B).

### Asymptomatic Patients Without an STI Co-infection Are More Frequently Characterized by Low-Diversity, *Lactobacillus*-Dominant Genital Communities

**FIGURE 4:**
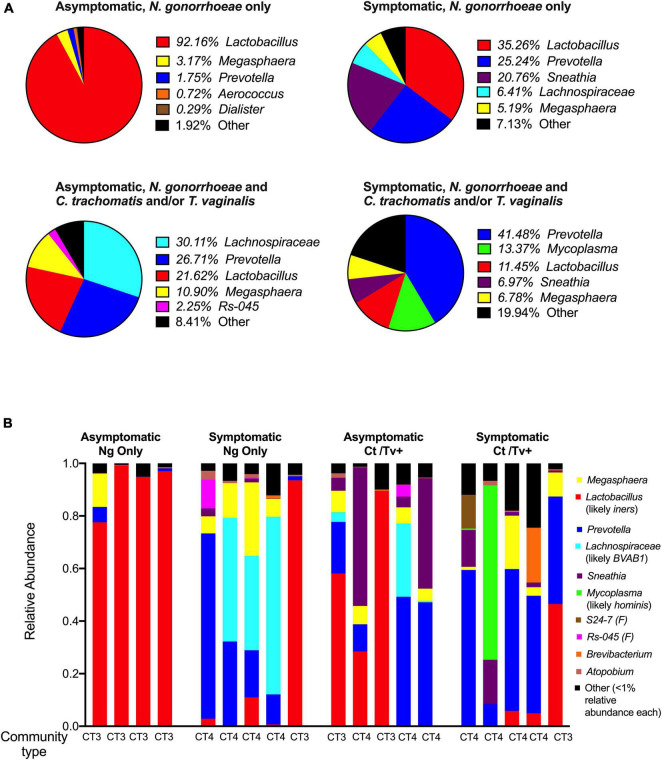
Community composition of symptomatic and asymptomatic individuals with and without *C. trachomatis* and/or *T. vaginalis* co-infection. The mean relative abundances of the top five most prevalent bacterial genera identified within each patient group of interest are plotted as pie charts with each pie representing a group of interest **(A)**. The relative abundances of the top ten taxa (genera- or family-level taxa, as applicable) identified across all 16S rRNA sequencing reads in the dataset are shown for each of the 19 participants included in the study. These top 10 taxa comprised ł 99% of all reads in the entire dataset. We used BLAST and MUSCLE alignments to determine the likely species of communities dominated by *Lactobacillus*, *Lachnospiraceae*, and *Mycoplasma* and found them to be *L. iners*, Bacterial Vaginosis-Associated Bacterium-1 (BVAB1)/“*Candidatus* Lachnocurva vaginae,” and *M. hominis*, respectively. The microbial community type (CT) designated for each participant, using standard definitions in the field, is provided **(B)**.

**FIGURE 5:**
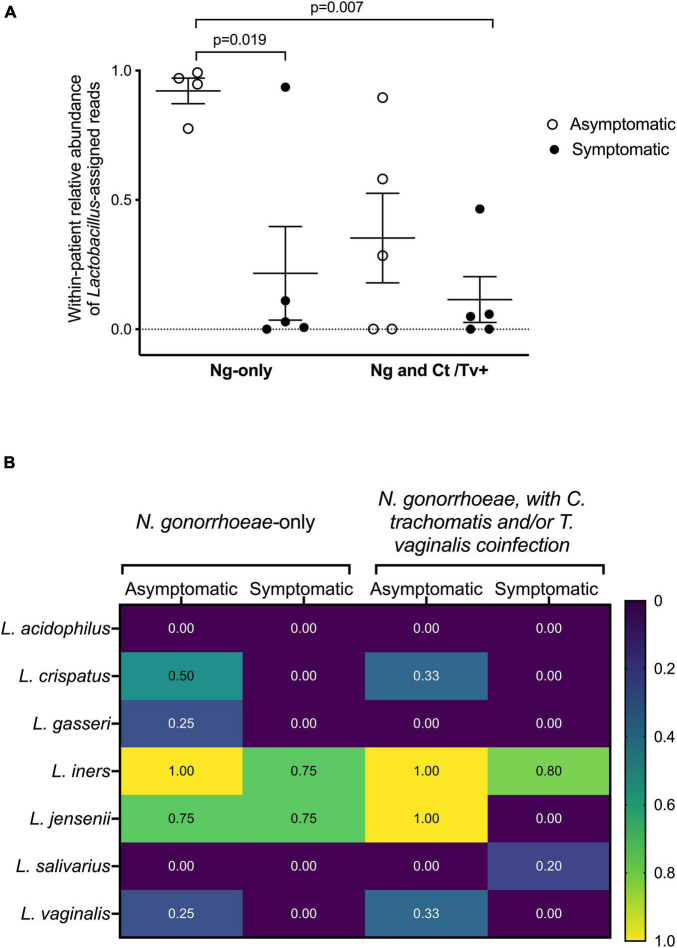
*Lactobacillus* spp. detection with respect to clinical presentation and other STI co-infection. The fraction of reads assigned to *Lactobacillus* among all sequence reads comprising an individual’s microbial community is shown **(A).** Participants have been grouped by symptom and co-infection status. Statistically significant differences between patient groups were explored with one-way ANOVA with Tukey’s correction for multiple comparisons. The presence of specific *Lactobacillus* spp. was also assessed by the presence/absence of commercially available real-time PCR assay **(B).** Participants have been grouped by symptom and co-infection status. The fraction of individuals within each group positive for each of the seven *Lactobacillus* species in our panel (*L. acidophilus*, *L. crispatus, L. gasseri, L. iners*, *L. jensenii*, *L. salivarius*, and *L. vaginalis*) are shown as a heatmap, with values ranging from “0” (i.e., none of the women in the group positive for that species) to “1.0” (i.e., all women in the group positive for that species).

The relative abundance of *Lactobacillus* spp. assigned reads differed by patient group. The distribution of *Lactobacillus* spp. relative abundances was as follows: 92.2% among asymptomatic individuals with no co-infection, 35.3% among asymptomatic individuals with co-infection, 21.6% among symptomatic individuals with no co-infection, and 11.5% among symptomatic individuals with co-infection (Figure 4A). This was evident when inspecting the individual taxa plots (Figure 4B), as the four asymptomatic patients with only *N. gonorrhoeae* infection and no detected co-infection were dominated by *Lactobacillus* taxa, whereas *Lactobacillus*-predominance was observed less frequently in specimens from women with symptomatic *N. gonorrhoeae* infection regardless of the presence of additional STI (2/10, 20.0% symptomatics vs. 6/9, 66.7% asymptomatics, *p* = 0.040, Figure 4A). Differences in within-patient relative abundance of *Lactobacillus* spp. in symptomatics with gonorrhea only vs. asymptomatics with gonorrhea were statistically significant (*p* = 0.019, Figure 5A). Similarly, the within-patient relative abundance of *Lactobacillus* spp. in symptomatics with gonorrhea only vs. that of symptomatic with co-infections also varied significantly (*p* = 0.007, Figure 5A).

Using BLAST and MUltiple Sequence Comparison by Log Expectation (MUSCLE) (Edgar, 2004), we investigated the 16S sequences of each taxa that was assigned *Lactobacillus* taxonomy and identified *L. iners* as the likeliest species (Supplementary Table 1). This led us to define *Lactobacillus*-dominant samples (*n* = 8) as community-type 3 (CT3), using standard definitions of vaginal microbial structure (Ravel et al., 2011; Anahtar et al., 2015; Gosmann et al., 2017; Figure 4B).

The presence of specific *Lactobacillus* species was further assessed by presence/absence real-time PCR (Figure 5B). The *Lactobacillus* species most commonly detected among all women were *L. iners*, *L. crispatus*, *L. jensenii*, and *L. gasseri* (Figure 5B). In line with our microbiome analyses and BLAST homology searches, all asymptomatic women were positive for *L. iners*, and in 85 and 75% of symptomatic women with and without co-infection, respectively. Although *L. crispatus* was not the predominant *Lactobacillus* species in any of the specimens using 16S sequencing, *L. crispatus* was detected by real-time PCR only in *N. gonorrhoeae*-infected individuals with asymptomatic presentation (Figure 5B).

### Symptomatic *Neisseria gonorrhoeae* and STI Co-infection Are Associated With a Diverse Cervicovaginal Microbial Community Composed of Bacterial Vaginosis-Associated Bacteria

Having established that 8 of 19 samples (42.1%) were *L. iners*-dominated (CT3) and more commonly associated with asymptomatic *N. gonorrhoeae* infection, we investigated in more detail the remaining 11 samples, which were dominated by a diverse group of non-lactobacilli (*Prevotella*, a *Lachnospiraceae* genus, *Sneathia*, or *Mycoplasma*). We used BLAST and MUSCLE alignments to further characterize the composition of the non-*Lactobacillus* communities dominated by *Lachnospiraceae* and *Mycoplasma*. By applying BLAST on representative reads assigned to each genus of interest, we found that the likeliest species for *Lachnospiraceae* and *Mycoplasma* OTUs were Bacterial Vaginosis-Associated Bacterium-1 (BVAB1)/“*Candidatus* Lachnocurva vaginae” and *M. hominis*, respectively (Supplementary Table 1). Samples dominated by *Prevotella* (*n* = 6), *Sneathia* (*n* = 1), BVAB1 (*n* = 3), and *M. hominis* (*n* = 1) were classified as CT4/molecular BV. The *Mycoplasma*-dominant sample was included in the CT4 and molecular BV classifications. The CT4/molecular BV samples were more frequently found in symptomatic patients (8/10, 80% of symptomatics vs. 3/9, 33.3% of asymptomatics, *p* = 0.040) (Figure 4B). Among STI co-infected individuals, 7/10 (70.0%) carried CT4 microbial communities compared to patients without co-infections (4/9, 44.4%), though this difference did not attain statistical significance (*p* = 0.255).

**FIGURE 6:**
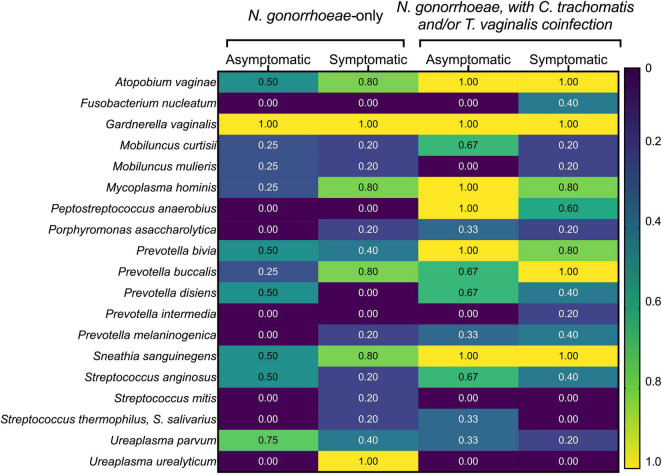
Prevalence of common bacterial vaginosis-associated bacteria detected with commercially available microbial DNA real-time PCR assay. The presence of the indicated BV-associated bacterial species was assessed by the presence/absence of commercially available real-time PCR assay. Participants have been grouped by symptom and co-infection status. The fraction of individuals within each group positive for each of the seven BV-associated bacteria in the panel are shown as a heatmap, with values ranging from “0” (i.e., none of the women in the group positive for that species) to “1.0” (i.e., all women in the group positive for that species).

The prevalence of common BV-associated bacteria was also assessed by commercially available microbial DNA real-time PCR assay (Figure 6). Of the BV-associated species included in the panel, *Gardnerella vaginalis* was present in all samples. Other species commonly associated with BV, like *Atopobium vaginae*, certain *Prevotella* spp., and *Sneathia sanguinegens* were also highly prevalent among this cohort of women, regardless of symptoms or STI co-infection status. Asymptomatic women infected only with *N. gonorrhoeae* (*n* = 4) carried the following species less frequently than symptomatic women infected only with *N. gonorrhoeae* (*n* = 5): *M. hominis* (25% vs. 80%), *Prevotella buccalis* (25% vs. 80%), and *Ureaplasma urealyticum* (0% vs. 100%). Similarly, asymptomatic females without co-infections also carried BV-associated bacteria less frequently when compared to those with symptoms or STI co-infections (Figure 6).

## Discussion

A large body of evidence links vaginal dysbiosis, such as clinical BV, to the risk of acquisition of several STIs, including gonorrhea (Martin et al., 1999; Atashili et al., 2008; Brotman, 2011; Bautista et al., 2017; Tamarelle et al., 2019). Despite the clear association between BV and STI acquisition risk, treatment of asymptomatic BV has not been found to reduce the incidence of *N. gonorrhoeae* or *C. trachomatis* infection incidence, raising the question whether a suboptimal vaginal environment is a modifiable biological cause of gonorrhea risk (Schwebke et al., 2016). However, *Lactobacillus*-based live probiotic therapy of vaginal dysbiosis has been recently shown to reduce not only BV (Aroutcheva et al., 2001; Witkin and Linhares, 2017), but also bacterial STI incidence (van de Wijgert and Verwijs, 2020). To the best of our knowledge, this report is the first to examine the association between the cervicovaginal microbiota composition and symptomatic *N. gonorrhoeae* infections in women. Using 16S ribosomal RNA gene deep sequencing approaches on patient samples confirmed to be infected with *N. gonorrhoeae* by Aptima clinical testing, we show that the cervicovaginal microbiome is predictive of gonorrhea clinical presentation in women attending an STI clinic in the United States. These findings were confirmed by real-time polymerase chain reaction assays specific for several *Lactobacillus* species and BV-associated bacteria deployed in parallel on the same clinical samples.

Specimens collected from asymptomatic individuals with *N. gonorrhoeae* infection and no co-infection with *Chlamydia trachomatis* and/or *Trichomonas vaginalis* carried *Lactobacillus-*dominant microbial communities more frequently than symptomatic patients without co-infection. Notably, this *Lactobacillus* dominance was due to *L. iners*, and these microbiotas were classified as community type 3 (CT3), according to established definitions in the field (Ravel et al., 2011; Anahtar et al., 2015; Gosmann et al., 2017). Previous studies have established that *L. iners*-dominated vaginal microbiotas compared to *L. crispatus*-dominated vaginal microbiotas may place patients more at risk of STI infection, like chlamydia or HIV (Gosmann et al., 2017; van der Veer et al., 2017; Tamarelle et al., 2019). Interestingly, none of the females in our study had cervicovaginal microbiomes dominated by *L. crispatus*, which may be consistent with a protective effect by *L. crispatus* on STI infection risk. This is supported by *in vitro* studies with clinically isolated and lab strains of *L. crispatus* have been shown to inhibit the growth of *N. gonorrhoeae in vitro* (Vielfort et al., 2008), possibly through the effects of lactic acid acidification of the growth environment (Graver and Wade, 2011). While *L. crispatus* produces both isomers of lactic acid, *L. iners* and human cells only make l(+) lactic acid (Spurbeck and Arvidson, 2010; Tachedjian et al., 2017). Accumulating evidence also suggests that d(−) lactic acid may impart greater protection against STI pathogens than l(+) lactic acid potentially *via* effects on human host cells rather than pathogen cells (Nunn et al., 2015; Edwards et al., 2019). In humans, *L. crispatus*-dominant vaginal microbiota is associated with reduced risk of acquisition of other STI, like HIV (Borgdorff et al., 2014; Gosmann et al., 2017; van der Veer et al., 2017; Tamarelle et al., 2019).

*Neisseria gonorrhoeae*-infected patients who reported symptoms were found to have genital microbiomes composed of a mixture of various bacterial anaerobes, such as *Prevotella*, *Sneathia*, *Mycoplasma hominis*, and BVAB1/“*Candidatus Lachnocurva vaginae*” (Holm et al., 2020). These women with genital microbiomes composed of anaerobes were deemed to have molecular BV, as defined by established classifications in the field based on diversity and relative abundance of bacterial taxa (Ravel et al., 2011; Anahtar et al., 2015; Gosmann et al., 2017). This included the *Mycoplasma*-dominant sample because of three main reasons that definitions of molecular BV take into account: like *Prevotella* and *Sneathia*, it can overgrow in cases of BV (Fredricks et al., 2005; Onderdonk et al., 2016), its prevalence in BV patients is three times higher compared to healthy women (Rumyantseva et al., 2019) and it is associated with severe genital mucosal inflammation (Martin et al., 2013).

A possible explanation for the association of symptoms in *N. gonorrhoeae* infection and BV-associated microbial communities relates to the known increase of inflammation and inflammatory mediators in women with BV. Several studies have shown that females with clinical BV or low *Lactobacillus* abundance and high diversity of anaerobes also harbor higher concentrations of pro-inflammatory cytokines in their genital tract (Anahtar et al., 2015) and higher levels of the pro-inflammatory cytokines (IL-1α, IL-1β, IL-6, IL-12, and IL-8) when compared to BV-negative women (Kyongo et al., 2015). Furthermore, symptoms of abnormal vaginal discharge were also found to associate with elevated levels of IL-1β, IP-10, IL-8, and GCSF, linking inflammatory cytokines to vaginal symptoms, particularly vaginal discharge (Kyongo et al., 2015). Notably, the more the vaginal microbiota shifts toward dysbiosis, the more marked the inflammation (Cohen et al., 2010; Anahtar et al., 2015; Lennard et al., 2018), independently of concurrent STIs, including HIV and gonorrhea (Anahtar et al., 2015).

We recognize that our study had limitations. Because of the nature of the study design, we had no information on whether our symptomatic subjects also had clinically defined BV. However, the association between BV-associated microbes and cervicovaginal community type suggests that further studies of the association of BV with symptomatic *N. gonorrhoeae* infection are needed. Because we lacked specimens from *N.* gonorrhoeae-negative women, our study was limited to analyses among *N. gonorrhoeae*-positive women. Thus, despite a strong association between low-*Lactobacillus* vaginal communities (molecular BV) and risk of acquisition of STIs like HIV and chlamydia (Borgdorff et al., 2014; Gosmann et al., 2017; van der Veer et al., 2017), as well as a strong association between clinical BV and risk of *N. gonorrhoeae* infection (Brotman, 2011; Gallo et al., 2012; Bautista et al., 2017), our study could not examine whether the vaginal microbiota composition associates in characteristic ways with risk or protection from *N. gonorrhoeae* infection. Instead, we focused on characterizing the relationship between vaginal communities and clinical presentation among women with clinically diagnosed *N. gonorrhoeae* infection. Future prospective studies are needed to determine the protective effect of Lactobacillus-dominated vaginal composition against *N. gonorrhoeae* infection. Finally, the majority of our study participants identified as African American. Previous BV prevalence studies reported that compared to Caucasian women, low-*Lactobacillus* vaginal microbiotas are more common in African American and Latin women (Zhou et al., 2007; Ravel et al., 2011; Fettweis et al., 2014) and that up to 50% of African American women may harbor vaginal microbiotas deplete in *Lactobacillus* species (Allsworth and Peipert, 2007). Future studies on how race, the vaginal microbiota, and *N. gonorrhoeae* risk intersect are needed.

 Overall, our study showed that symptomatic vs. asymptomatic gonorrhea presentation is correlated with having molecular BV, leading to two possible explanations. First, that the molecular BV community type compared to the *L. iners* dominated community type may have predisposed to the development of gonococcal-associated symptoms. Second, that the BV state developed after or was even caused by the establishment of *N. gonorrhoeae* infection due to the promotion of the growth of BV-associated bacteria or a loss of *Lactobacillus* species. Thus, our findings suggest that the cervicovaginal microbiota is a determinant, or at least a contributor, to gonorrhea clinical presentation in women. Further studies defining the relationship between genital tract microbiomes and the pro-inflammatory immune responses in symptomatic presentation of *N. gonorrhoeae* infection are needed to elucidate whether *Lactobacilli* or BV-defining microbial communities serve as a biomarker for symptoms in *N. gonorrhoeae* infections or directly impact symptoms.

## Data Availability Statement

The datasets presented in this study (accession PRJNA768436) can be found at the link: https://www.ncbi.nlm.nih.gov/bioproject/?term=%20PRJNA768436.

## Ethics Statement

The studies involving human-derived specimens were reviewed and approved by the University of North Carolina Institutional Review Board (Studies 11-0047 and 15-2531). The research was found to meet the criteria for a waiver of informed consent for research [45 CFR 46.116(d)] and waiver of HIPAA authorization [45 CFR 164.512(i)(2)(ii)] as the study entailed research on existing specimens, posing minimal risk to participants; the waiver did not adversely affect the rights or welfare of the participants, and consent/assent would have been impracticable given the loss to follow up.

## Author Contributions

All authors contributed to the writing and revision of the manuscript. AL wrote the first draft of the manuscript, performed the lab investigation, and analyzed the data. JD conceptualized the study. JD and AW conducted formal analysis, methodology selection and experimental design, and supervision. AS oversaw clinical specimen collection. GS and AM contributed to experimental design and data analysis.

## Conflict of Interest

The authors declare that the research was conducted in the absence of any commercial or financial relationships that could be construed as a potential conflict of interest.

## Publisher’s Note

All claims expressed in this article are solely those of the authors and do not necessarily represent those of their affiliated organizations, or those of the publisher, the editors and the reviewers. Any product that may be evaluated in this article, or claim that may be made by its manufacturer, is not guaranteed or endorsed by the publisher.
