# Supplementary figures and images for: Cervicovaginal Microbiota Predicts Neisseria gonorrhoeae Clinical Presentation
Source: Front Microbiol. 2022 Feb 10;12:790531. doi: 10.3389/fmicb.2021.790531 (PMC8867028; doi:10.3389/fmicb.2021.790531)

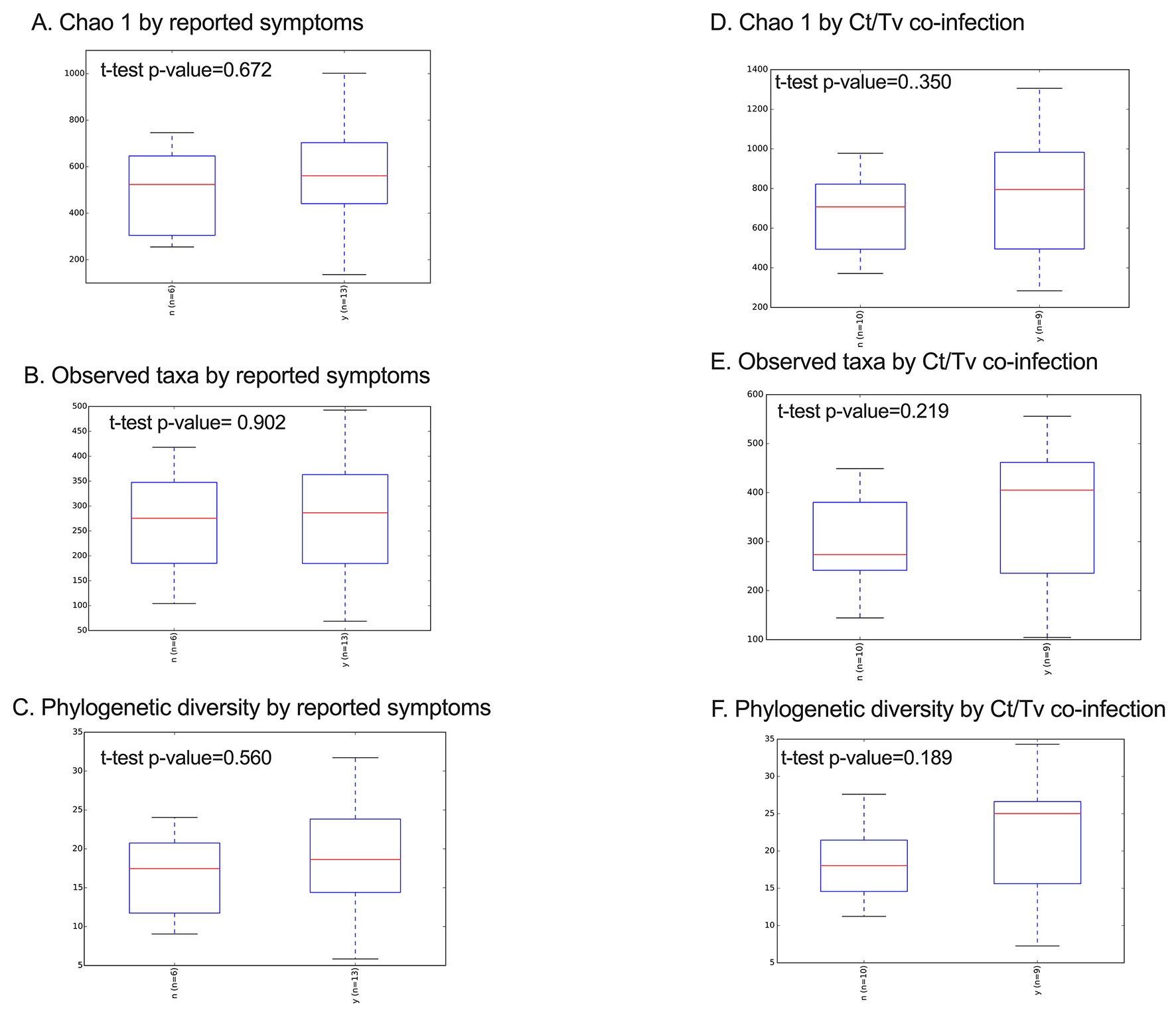

Supplement: Supplementary file 1 [file Image_1.TIFF]
